# How to deal with non-detectable and outlying values in biomarker research: Best practices and recommendations for univariate imputation approaches

**DOI:** 10.1016/j.cpnec.2021.100052

**Published:** 2021-03-29

**Authors:** Judith Herbers, Robert Miller, Andreas Walther, Lena Schindler, Kornelius Schmidt, Wei Gao, Florian Rupprecht

**Affiliations:** a Faculty of Psychology, Technische Universität Dresden, Dresden, Germany; b Health Technology Assessment & Outcomes Research, Health & Value, Pfizer, Germany; c Clinical Psychology and Psychotherapy, University of Zurich, Switzerland

**Keywords:** Limit of quantification, Non-detectables, Outlier, Imputation, Best practices, Simulation study

## Abstract

Non-detectable (ND) and outlying concentration values (OV) are a common challenge of biomarker investigations. However, best practices on how to aptly deal with the affected cases are still missing. The high methodological heterogeneity in biomarker-oriented research, as for example, in the field of psychoneuroendocrinology, and the statistical bias in some of the applied methods may compromise the robustness, comparability, and generalizability of research findings. In this paper, we describe the occurrence of ND and OV in terms of a model that considers them as censored data, for instance due to measurement error cutoffs. We then present common univariate approaches in handling ND and OV by highlighting their respective strengths and drawbacks. In a simulation study with lognormal distributed data, we compare the performance of six selected methods, ranging from simple and commonly used to more sophisticated imputation procedures, in four scenarios with varying patterns of censored values as well as for a broad range of cutoffs. Especially deletion, but also fixed-value imputations bear a high risk of biased and pseudo-precise parameter estimates. We also introduce censored regressions as a more sophisticated option for a direct modeling of the censored data. Our analyses demonstrate the impact of ND and OV handling methods on the results of biomarker-oriented research, supporting the need for transparent reporting and the implementation of best practices. In our simulations, the use of imputed data from the censored intervals of a fitted lognormal distribution shows preferable properties regarding our established criteria. We provide the algorithm for this favored routine for a direct application in R on the Open Science Framework (https://osf.io/spgtv). Further research is needed to evaluate the performance of the algorithm in various contexts, for example when the underlying assumptions do not hold. We conclude with recommendations and potential further improvements for the field.

## Highlights

• ND and OV are considered as censored data, e.g. due to measurement error cutoffs.
• Several common univariate approaches in handling ND and OV are presented.
• In a simulation study, their performances are compared.
• A novel algorithm shows preferable properties.
• General recommendations on how to deal with ND and OV are presented.

## 1 Introduction

Biopsychological research has been working for many years to identify reliable and valid biomarkers in order to improve the understanding, diagnosis and treatment of psychological conditions [1]. Due to the ongoing research progress, biomarkers (e.g. steroid hormones like cortisol) can now be determined in a plethora of specimens – from plasma and saliva for the assessment of current secretion to urine or hair sampling for the retrospective investigation of longer-term processes [[2], [3], [4]]. In order to better comprehend the complex interplay of physiological and psychological processes, the trend goes toward concurrent analyses of multiple biomarkers [5,6]. While there already are efforts to standardize sampling and laboratory procedures (e.g. [3,[7], [8], [9], [10], [11]]), to the best of our knowledge, so far no such consensus exists on how to deal with non-detectable (ND) and outlying value concentrations (OV). With this paper, we intend to provide general recommendations for the handling and reporting of ND and OV in biomarker research with emphasis on univariate imputation approaches.

When methods from analytical chemistry are used, ND and OV often arise from data which is partially unknown (i.e., censored) due to a restricted sensitivity and precision of the applied assays [[12], [13], [14]]. They present a regular challenge for biomarker research, where they can amount to particularly high proportions of up to 50% ([15]; but see also [16] and [13]). In order to ensure statistical validity of consecutive analyses, such as analyses of variance (ANOVA) or linear regression, these data need to be considered and modeled properly – either during data preprocessing, or within the analysis procedure itself.

In the field, various methods are applied to handle ND and OV. Their complexity ranges from the case-wise deletion of affected data (basically treating them as “missing”), or single imputation to the use of multiple imputation and the application of censored regression models like Tobit models. Previous research has already demonstrated that case-wise deletion and single imputation are outperformed by more sophisticated methods like multiple imputation due to bearing a high risk of biased parameter estimates, especially for high amounts of affected cases (e.g. [[17], [18], [19], [20]]), and an inflated number of false-positive results [16]. However, these simple methods are still popular in the field. An exploratory screening of all openly available articles published in *Psychoneuroendocrinology* in 2019 (N ​= ​48) suggested the prevailing application of exclusion and single imputation techniques for both ND and OV. Notably, the considerable dispersion of this estimate was due to the often opaque or lacking reporting of the applied methods to handle ND and OV (for more details on our exploratory screening, see Supplementary Material S1). Different reasons can be imagined for the low penetrance of demonstrably better methods, such as suboptimal technical know-how to implement the sophisticated methods, or a lack of incentives to specify complex imputation models. Assuming that both explanations may be valid, we will focus on considerations regarding easy-to-implement and practical methods for handling ND and OV in this article.

In the following, we aim to present and compare common methods in dealing with ND and OV and derive general best practices for biomarker-oriented research. This goal is divided into four steps: First, we introduce a generic model of biochemical measurement methods, demonstrating how measurement imprecision drives both ND and OV. Second, we provide an overview on common univariate approaches to handle ND and OV by giving a short description including the advantages and drawbacks for each method.^1^ Third, we perform a simulation study on a model data set. Here, we compare the performance of deletion and five different imputation-based methods for ND and OV. The code, simulated data and the R package with the proposed algorithm can be accessed at https://osf.io/spgtv/. Fourth, we present censored regression models (e.g. Tobit models) as an advanced option for handling censored data structures and conclude with general recommendations for the improvement of quality standards in the field.

## 2 A generic model of biochemical measurement methods

In the following section, we will introduce a generic model of biochemical quantification that illustrates the joint characteristic of measurement imprecision for ND and OV. The fundamental component of this model are limits of quantification (LOQ).^2^ Typically, an assay, e.g. Liquid Chromatography Tandem Mass Spectrometry (LC-MS/MS), has both a lower limit of quantification (LLOQ) and an upper limit of quantification (ULOQ), which mark the endpoints of an operational range where measurements can be reliably performed. Laboratories often report values below the LLOQ as ND and might mark values above the ULOQ as OV. The subsequent sections will explore in more detail where these limits come from and how they can be obtained.

### 2.1 Calibration curve and coefficient of variation

In biomarker research, the parameter of interest is typically a concentration estimate of the analyte, e.g. the cortisol concentration in a saliva sample. However, initially, the analytic tool returns a value of signal strength, which then this is then rescaled to a concentration value according to a calibration or standard curve. Such a curve is constructed from repeatedly measured calibrators with known amounts of concentration (i.e. spiked samples) in a standardized fashion. A fit of known concentrations with corresponding signal strengths leads to a signal-concentration calibration curve for the applied calibrator range. Ideally, this applied calibrator range covers the entire range of expected concentrations [21,22].

**Fig. 1:**
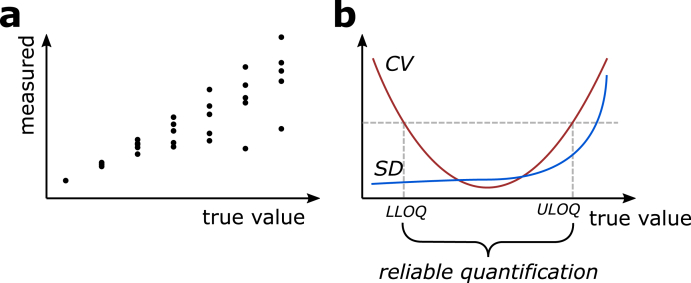
Visualization of the measurement model. a) Schematic visualization of exemplary signal-to-concentration data. In this example, data is available for seven concentrations with five samples, respectively. b) Schematic representation of the emergence of quantification limits based on the coefficient of variation (CV) curve. Notably, the CV is greater than zero for each concentration, but will increase approaching both ends of the measured range. It is now to determine when a measurement is declared unreliable, i.e. at which points the CV exceeds a measurement error cutoff, e.g. 10%. These cutoffs are the lower and upper limit of quantification (LLOQ and ULOQ).

Additionally, the observed variability of signal strengths from identical calibrator concentrations serve as index of precision, expressed e.g. as coefficient of variation (CV). The CV is a ratio of the standard deviation (SD) of the measured signal strengths $\sigma \in R_{\geq 0}$ to the respective mean $\mu \;\in R_{>0}$, $CV\;=\;\frac{\sigma }{\mu }$ . It allows for a quantification of the measurement error in percent, independent of unit or range [23]. A simple illustration for a signal-to-concentration data and the resulting CV curve is presented in Fig. 1.

The range of reliable quantification is that in which the CV lies below an accepted value. Recommended cutoffs for the CV in bioanalytical method validation are e.g. 10% or 20% [17,24]. An exceedance of the applied cutoff at the lower end marks the LLOQ, and, conversely at the upper end, the upper limit of quantification (ULOQ) [21,22]. In other words, the LLOQ corresponds to the lowest and the ULOQ to the highest concentration that can be quantified with acceptable analytical precision [25,26]. The working or operational range lies in between these cutoffs. It is to mention that we focus on the aspect of measurement precision for the purpose of this paper. Importantly, the working range of an assay has some additional properties, and we recommend the reading of Shah et al. [22] and Lee et al. [21] for a more elaborated consideration.

### 2.2 Cutoff criteria

The determination of unreliable data requires appropriate criteria. A convenient way to do so is using cutoff limits and define both samples below a lower cutoff (LC) and above an upper cutoff (UC) as unreliable. At part, this is already implicitly done for samples below the limit of detection (LOD, see glossary) when they are marked as ND. Ideally, the LLOQ and ULOQ derived from the precision profile are used as cutoffs. However, other criteria may also be considered. This could be for example reference ranges outside which values are considered biologically implausible. Regarding OV, cutoff criteria might also include certain distance rules, e.g. +2.5 or + 3SD [27,28].^3^

### 2.3 Data distribution in biomarker research

Distributions of biomarker data, e.g. of saliva cortisol concentrations, are reported to be right-skewed and leptokurtic [29]. A distribution that matches these observed characteristics of biomarker data is the lognormal distribution, which we will assume to be the data-generating distribution underlying biomarker data. However, in practice, there may be cases in which the data-generating distribution varies from that assumption, for instance in regard to the skewness, which may be less [30] or more pronounced than that of a lognormal distribution. The presumed data-generating distribution may vary in shape and in its position in the operational range of the assay. In the case of an investigation where this distribution is proximate to a LC (e.g. the LLOQ), many truly low concentrations will fall below this cutoff (BC ​< ​LC). They might be marked as ND because they are not only below the LC, but also below the LOD. Although the exact values of these samples may be unknown, they are not missing at random (MNAR; for an overview over other types of missing data, see e.g. [31] over the whole range of the biomarker). Instead, the fact that they are missing can be considered informative, because they reflect truly low concentrations of the respective biomarker [13,14]. An LC would therefore induce left-censoring and skewness to the data, whereby the left-censoring limit would be equal to the LC.

In another investigation, the distribution may lie proximate to an UC (e.g. the ULOQ). Thus, many true concentrations will be above this cutoff (AC ​> ​UC). Here, an UC would induce right-censoring to the data, whereby the censoring limit would be equal to the UC. For a better overview, we summarized the used abbreviations and a short definition of each in Table 1.

**Table 1:** Glossary of used abbreviations and corresponding definitions.

Abbreviation	Term	Definition
LOD	limit of detection	lowest concentration that is significantly different from background noise (e.g. a blank sample [21])
LOQ	limit of quantification	limit at which the assay is able to provide quantitative results of a stated analytical quality, e.g. precision [25]. Often, both a lower and upper limit exist.
LLOQ	lower limit of quantification	lowest concentration at which the assay provides quantitative results of a stated analytical quantity, e.g. precision
ULOQ	upper limit of quantification	highest concentration at which the assay provides quantitative results of a stated analytical quantity, e.g. precision
LC	lower cutoff	lower cutoff in the model, below which all data becomes censored
UC	upper cutoff	upper cutoff in the model, above which all data becomes censored
BC	below (lower) cutoff	data below the lower cutoff
AC	above (upper) cutoff	data above the upper cutoff
CV	coefficient of variation	deviation of values in relation to the respective mean
ND	non-detectable	concentrations below the LOD, definitions may vary (see also [33])
OV	outlying value	implausible high concentration, definitions may vary

It is therefore crucial that researchers consider if their data is reliably or unreliably quantified to ensure reliability and validity of the consecutive data analyses. During data processing, ND and OV then need appropriate handling or modeling to guarantee successful future efforts in identifying clinically reliable biomarkers [32].

## 3 Common univariate handling approaches for ND and OV

Being confronted with a high proportion of samples BC (including ND) and AC (including OV) as typical for biomarker research leads to the question of how to aptly deal with these samples. Common techniques can be mainly distinguished in (1) deletion of all affected cases and (2) substitution using different imputation-based approaches, both in order to process the data for consecutive statistical analyses. We hereby focus our considerations of these techniques in univariate settings, by which we mean either contexts, in which only one biomarker is measured, or analyses, in which only a single biomarker is considered.

### 3.1 Deletion

One simple and straightforward solution that could come to mind is to exclude all samples BC and AC, which would equal a case-wise deletion and thus a complete-cases analysis [34]. The procedure is analogous to a trimming procedure for OV [35,36].

The apparent advantage of this method is its simplicity. However, tempting as it therefore may be, researchers must bear in mind that deletion has at least two major disadvantages: First, the exclusion of cases decreases the sample size and, thus, the statistical power [34,37]. Second, limiting the analyses on the uncensored data necessarily leads to systematic bias. In the presence of samples BC, the resulting data distribution is consequently left-censored and will become (more) skewed. In the presence of samples AC, this will lead to an additional right-censoring and skewing. This is because the measured values are not representative for the censored values, so their absence would bias the parameter estimates [13].

However, a trimming procedure may be the appropriate choice for OV if it is assumed that they are the consequence of some error or undesired effect (e.g. sample contamination [27]). In the case of legitimate measurements, however, the same issues as for samples BC and AC arise.

### 3.2 Imputation-based approaches

Another way to handle samples BC and AC is the use of imputation, i.e., the replacement of affected cases with substituted values. We distinguish two forms of imputation: The imputation of a fixed value and the imputation of values sampled from a distribution.

#### 3.2.1 Fixed-value imputation

Fixed-value imputation corresponds to a replacement of each sample BC and AC with a fixed value. Fixed-value imputation excels deletion because no cases are excluded and therefore sample size remains unaffected. Regarding BC, several procedures exist, some of which are based on the observed data [37] and others derived from the applied measurement instrument [14].

One example for a fixed-value imputation based on the observed data is the overall mean imputation, where samples BC and AC are replaced with the mean of the observed values [31]. A related approach is the imputation of the median of the observed values. However, mean or median imputation are only appropriate if they are representative for the censored cases – which directly contradicts the assumption that these samples are MNAR. Given this setting, mean or median imputation for samples BC and AC would therefore induce a systematic bias and underestimate variance [12], with both artifacts increasing as the number of imputed values increases.

An improvement that could come to mind is to substitute the affected samples with values related to the quantification limits or limits of the measured range. For samples BC, commonly applied methods are, e.g. a replacement with LLOQ, LLOQ/2, or zero [14]. The first two methods hereby implicitly assume that the LC is equal to the LLOQ. Following the same logic, fixed-value imputations for samples AC are plausible. Substituted values could be, e.g. the ULOQ, the upper limit of the measured range or a combination of both. Fixed-value imputation is less conservative than trimming, as it preserves the relative ranking of AC samples. This procedure can also be seen related to winsorizing described in the literature on outlier handling, where OV above a certain cutoff are replaced by a more plausible value, for example the respective cutoff value [35,36]. Furthermore, as all cases remain in the analysis, essential information and sample size are preserved while potentially harmful effects as imprecise quantification are reduced. However, while these methods account for the assumed truly low and high concentrations, they still induce biased standard deviation estimates, simply because the imputed values lack any variance.

#### 3.2.2 Distribution-based imputation

Another possibility is to impute values from a distribution for the censored intervals. Such procedures allow the consideration of the assumed mean in these intervals while including some variance. They require assumptions on the underlying data-generating distribution. Using single imputation, each censored sample is replaced with a single value drawn from the assumed data-generating distribution. A method that additionally allows to account for the uncertainty that comes along with the between-imputation variability of missing data is multiple imputation, which outperforms single imputation e.g. in regard to the estimation of standard errors [38]; for a hands-on introduction, we recommend a reading of [18]. Therefore, multiple imputation should be preferred over single imputation. Multiple imputation can be easily achieved by repeating the imputation procedure multiple times, obtaining parameter estimates for each of the resulting imputed datasets and pooling these estimates according to Rubin’s rules (see Ref. [31]).

##### 3.2.2.1 Imputation from a uniform distribution

One possibility is to impute values from a uniform distribution for the censored intervals. By doing so, random draws in the interval [0, LC] for BC would result in a mean of LC/2 (and a variance of LC^2^/12) for the imputed values. Analogously for samples AC, values can be imputed from the interval between UC and e.g. the upper limit of the measured range. Though, as for fixed-value imputation, the limits of the measured range are not always known.

Hence, this approach assumes that the data follows a uniform distribution in the censored intervals, which, in reality, is an unlikely outcome. As biomarker distributions, e.g. hormone concentrations, are reported to be right-skewed [29,30], both the assumptions of a normal and uniform distribution are violated, which challenges the validity of this approach.

##### 3.2.2.2 Imputation from a fitted distribution

An improvement of the prior approach is to sample the censored values from a distribution that is fitted to all available data. This requires information on both the observed data as well as the amount and probability of the censored data to fall below or above the LC and UC, respectively. It also requires assumptions with respect to the properties of the data-generating distribution. The algorithm proposed here is a novel approach in which distribution parameters are estimated via maximum likelihood estimation for censored data. The algorithm identifies the underlying uncensored data distribution, in this case assuming a lognormal distribution, by incorporating the observed values as well as the number of samples BC and AC and the LC and UC (e.g. the LLOQ and the ULOQ). Substituting values for the censored samples can then be sampled from the truncated parts of the fitted distribution. Besides the advantage of a good fit, censored distribution fitting facilitates a uniform and simultaneous handling of both samples BC and AC. This approach is implemented in R using the fitdistrplus and EnvStats R packages [39,40]. All code can be accessed at https://osf.io/spgtv/.

## 4 Simulation study

### 4.1 Model and data sets

In order to demonstrate the influence of the applied methods for handling samples BC and AC, we created model data sets using R [41]. For all settings, the true values were randomly drawn from a lognormal distribution (such as is oftentimes the case for biological data, e.g. cortisol levels) with adjustable shape and location. The simulated model had four additional adjustable components, which are CV, calibrators, data distribution and cut-offs (i.e. LLOQ and ULOQ). More details on the model are provided in the Supplementary Material S2. We examined the performance of six methods regarding four criteria: (1) Differences in the estimates of mean, (2) median and (3) standard deviation between true and reconstructed data distribution as well as (4) the Kolmogorov-Smirnov distance (d_KS_) as a measure for the distance between the true data distribution and the reconstructed data distribution after deletion or imputation.

The six methods are:

• M_del_ Deletion
• M_fix1_ Imputation of the minimum and maximum from the measurement range for samples BC and AC, respectively
• M_fix2_ Imputation of the mean between zero and lower cut-off for samples BC and the mean between upper cut-off and upper range limit for samples AC
• M_fix3_ Imputation of the lower cutoff value for samples BC and the upper cut-off for samples AC
• M_uni_ Imputation for samples BC and AC from a uniform distribution in the respective intervals
• M_log_ Imputation for samples BC and AC from a fitted lognormal distribution in the respective intervals

Note that M_fix1_, M_fix2_ and M_fix3_ equal fixed-value imputations described in 3.2.1, while M_uni_ and M_log_ are distribution-based imputations described in 3.2.2. We used multiple imputation for the distribution-based imputations in this simulation study.

### 4.2 Performance in four scenarios

**Fig. 2:**
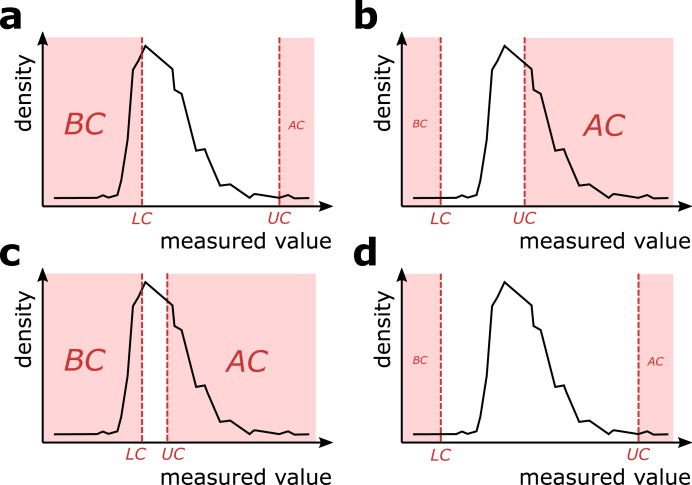
**Visualization of the four simulated scenarios.** They differ in the position of the distribution in respect to the cutoffs and the width of the censored intervals. a) Scenario with a high percentage of samples BC but little percentage of samples AC. b) Scenario with a low percentage of samples BC but a high percentage of samples AC. c) Scenario with both high percentages of samples BC and AC. d) Scenario with both low percentages of samples BC and AC.

First, we simulated the performance in four scenarios with exemplary settings resulting in distinct patterns of censored values of which we think they could be relevant for biomarker research (Fig. 2). (A) Scenario with a high percentage of samples BC but little percentage of samples AC. (B) Scenario with a low percentage of samples BC but a high percentage of samples AC. (C) Scenario with both high percentages of samples BC and AC. (D) Scenario with both low percentages of samples BC and AC. In all scenarios, the LC and UC were identical with the LLOQ and ULOQ, respectively. We observed deviations in the estimated mean, median, and standard deviation, as well as KS distance for the six applied methods. The parameter settings for each scenario are provided in the Supplementary Material S3. For each scenario, we ran the simulation with 50 samples and display the average outcomes over these simulation runs per scenario. The stability of the methods across a range of cutoffs will be further examined in the subsequent simulations.

#### 4.2.1 Scenarios

##### 4.2.1.1 Scenario A: High percentage of samples BC

In Scenario A, we chose the parameters in a way that the pattern of observed data represents true data that is distributed closer to the lower limit of the working range. The data sets consisted of approximately 26% samples BC and approx. 2% samples AC. In sum, this equals 28% of censored data. This amount of missing values is in line with previous reports [16], and a high prevalence of samples BC appears to be frequent in biomarker research [15,16,19].

##### 4.2.1.2 Scenario B: High percentage of samples AC

Conversely to Scenario A, in Scenario B we simulated a high percentage of samples AC and a low percentage of samples BC. Therefore, the true data is in closer proximity to the upper limit of the working range. Due to the cutoffs, the resulting data sets had approximately 1% samples BC and approximately 26% samples AC. In sum, approx. 27% of the data was censored, a comparable amount to Scenario A.

##### 4.2.1.3 Scenario C: High percentages of samples BC and AC

In Scenario C, we created data sets with both high percentages of samples BC and samples AC. Such data could occur as a result of widely distributed values in a sample that exceed the working range of the assay on both ends. It could also occur more generally if the range of expected values is not well covered by the working range. Here, in sum, our data sets consisted of approx. 67% censored values, approx. 47% of samples BC and 20% of samples AC.

##### 4.2.1.4 Scenario D: Low percentages of samples BC and AC

In Scenario D, we simulated the performance of the six presented methods in the presence of a low percentage of samples BC and AC. Scenario D is practically the most favorable because there is only a small amount of censored data which means that the operational range covers well the sample data. The resulting data sets consisted of 2% of samples BC and 2% of samples AC. In sum, approx. 4% of samples were censored.

#### 4.2.2 Results

Table 2 summarizes the results regarding all four scenarios (A to D) for the six applied methods. A deletion of the censored cases (M_del_) provided large differences between true data distribution and reconstructed data distribution for mean, standard and median estimates in all scenarios. This was also true in Scenario D with only a small percentage of censored values. Additionally, d_KS_ was highest for this method in three of four scenarios (A, B and D). The results in all four criteria for fixed value imputation (M_fix1_ – M_fix3_) were inconsistent, depending on the amount and pattern of censored values. d_KS_ was large for M_fix1_ – M_fix3_. M_uni_ and M_log_ produce less difference in the estimates for mean, standard deviation and median in all scenarios. For M_log_, d_KS_ distance was smallest in all scenarios. Overall, deviations in parameter estimates were larger for scenarios with higher percentages of censored values and lower for small percentages of censored values. A visualization is provided in the Supplementary Material S4.

**Table 2:** **Outcomes for the six applied methods in Scenarios A-D**. Average deviation in mean, standard deviation (SD) and median estimate of the reconstructed distribution and the true distribution in percent. d_KS_ is the average Kolmogorov-Smirnov distance between the distributions. The displayed results are the averages of 50 simulation runs per scenario.

	Δ mean (%)	Δ SD (%)	Δ median (%)	d_KS_
Scenario A				

M_del_	14.650	−25.694	23.011	0.261
M_fix1_	−7.596	26.009	0.100	0.261
M_fix2_	−2.783	2.980	0.100	0.218
M_fix3_	2.030	−17.088	0.100	0.257
M_uni_	−2.802	4.176	0.100	0.093
M_log_	−0.768	−4.782	0.100	0.021
Scenario B				
M_del_	−29.722	−60.869	−18.095	0.306
M_fix1_	20.797	49.172	−3.034	0.241
M_fix2_	0.916	−4.566	−3.034	0.186
M_fix3_	−18.966	−52.410	−3.034	0.304
M_uni_	0.775	2.152	−3.034	0.065
M_log_	−7.668	−20.475	−3.034	0.054

Scenario C				
M_del_	−11.819	−83.717	17.704	0.436
M_fix1_	1.472	51.889	−7.478	0.472
M_fix2_	−7.734	−12.898	−7.478	0.329
M_fix3_	−16.940	−75.921	−7.478	0.435
M_uni_	−7.886	−5.006	−7.478	0.114
M_log_	−17.095	−39.137	−7.478	0.087
Scenario D				
M_del_	−2.229	−15.501	−0.022	0.028
M_fix1_	1.719	25.077	0.107	0.022
M_fix2_	0.397	6.872	0.107	0.021
M_fix3_	−0.925	−7.302	0.107	0.023
M_uni_	0.374	7.736	0.107	0.019
M_log_	−0.275	−1.249	0.107	0.019

*Note.* Numbers closer to zero indicate a smaller difference between true data distribution and data distribution after deletion or imputation. The results come from exemplary parameter settings and the true estimates are not known in practical applications. Other settings with the same percent of censored values might lead to slightly different outcomes. M_del_: Deletion; M_fix1_: Imputation of the minimum and maximum from the measurement range for samples BC and AC, respectively; M_fix2_: Imputation of the mean between zero and lower cut-off for samples BC and the mean between upper cut-off and upper range limit for samples AC; M_fix3_: Imputation of the lower cutoff value for samples BC and the upper cut-off for samples AC; M_uni_: Imputation for samples BC and AC from a uniform distribution in the respective intervals M_log_: Imputation for samples BC and AC from a fitted lognormal distribution in the respective intervals.

### 4.3 Systematic variation of cutoffs

**Fig. 3:**
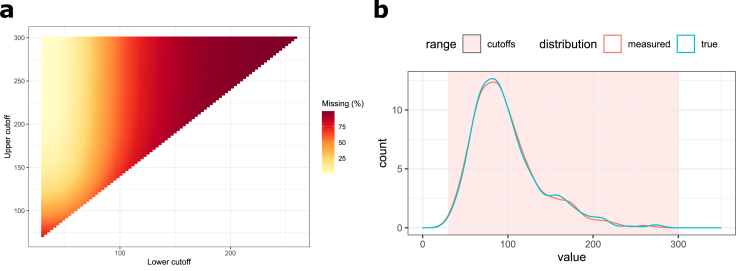
**Simulated lognormal data distribution, range of applied cutoffs and resulting proportions of missing data.** a) Shown are the true (green) and the measured data distribution (red), which results after applying the respective coefficient of variation (CV) to the true data distribution. LC and UC were in the range of [30,300], whereby the difference between UC and LC was at least 40 (arbitrary units). b) Proportions of missing values for each cutoff combination. Increasing color intensity corresponds to an increased proportion of missing values. (For interpretation of the references to color in this figure legend, the reader is referred to the Web version of this article.)

In the previous section, we have investigated the performance of the six applied methods in four exemplary scenarios (see Fig. 2). We have extended this simulation by examining the performance on a broad range of positions for LC and UC, resulting in various amounts and patterns of censored data (see Fig. 3). As in the previous section, we here provide the averaged outcomes of 50 simulation runs. Additional details on the simulations are provided in the Supplement S2 and S5.

#### 4.3.1 Trends for the mean

**Fig. 4:**
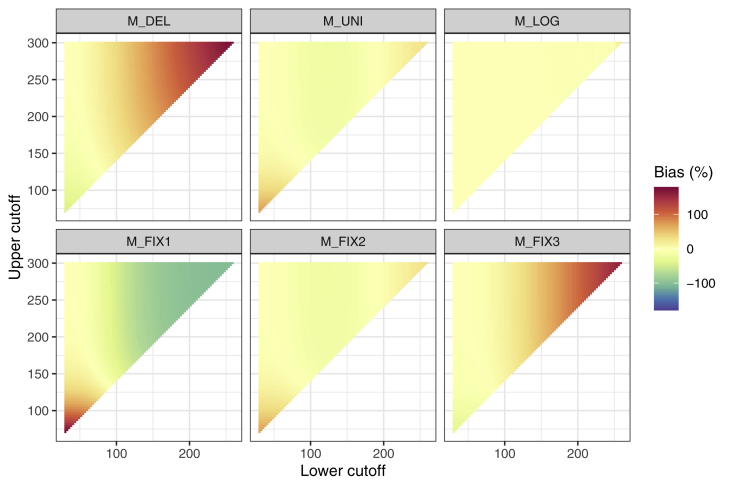
**Deviation of the mean estimate of the reconstructed distribution compared to the mean of the true distribution.** See for example the performance of method M_del_ (top-left) depending on both the LC and UC: LC increases from left to right. At the top, practically no censoring due to an UC is apparent, so that the isolated trend for an increasing LC can be observed. The higher the LC gets, the more it approaches the mean of the sample and the more cases are BC. Consequently, a deletion of the censored samples would lead to a large positive difference of the sample mean (increasing color intensity for red), whereby the amount is increasing for a rising LC. Conversely, a deletion of samples AC would lead to an underestimate of the sample mean, whereby the amount increases for a decreasing UC (i.e., in the direction of the sample’s mean). Combinations of LC and UC, for which the adverse effects of BC and AC deletion cancel each other out so that the mean difference is relatively small could potentially occur. However, in these scenarios, the standard deviation would still be underestimated (see 4.3.2). (For interpretation of the references to color in this figure legend, the reader is referred to the Web version of this article.)

Fig. 4 shows the results for the estimate of the mean for all compared methods. All in all, the trend for M_del_ is to overestimate the sample mean compared to the mean of the true data distribution in the presence of a high percentage of samples AC. In turn, the mean is underestimated for a high percentage of samples BC. The same trend can be observed for M_fix3_, even though the bias is overall smaller than for M_del_.

The patterns for method M_fix1_, M_fix2_ and M_uni_ are opposing. M_fix1_ and M_fix2_ incorporate the measurement range, which leads to an underestimate of the mean of the reconstructed distribution compared to the mean of the true data distribution for an increasing number of samples BC and to an overestimation of the mean for an increasing number of samples AC for both methods. This effect is stronger for M_fix1_ than for M_fix2_.

M_uni_ and M_log_ show only a minor difference in the mean of the reconstructed and true data distribution for all combinations of LC and UC. This is supported by the mean squared error (MSE) for each method over all possible cutoff combinations, which is smallest for method M_log_ (Table 3).

**Table 3:** *Outcomes for the six applied methods in the simulation with systematic cutoff variation.* Average deviation (50 simulation runs) in mean, standard deviation (SD) and median of the reconstructed distribution and the true distribution in percent. *d*_*KS*_ is the average Kolmogorov-Smirnov distance between the distributions.

Method	Δ mean (%)	Δ SD (%)	Δ median (%)	d_KS_
M_del_	41.089	−37.830	46.342	0.536
M_fix1_	−34.892	70.073	−49.744	0.536
M_fix2_	−2.984	10.876	−8.836	0.406
M_fix3_	28.924	−43.665	32.073	0.536
M_uni_	−2.987	42.076	−1.500	0.183
M_log_	0.214	−0.840	0.311	0.057

*Notes.* Numbers closer to zero indicate a smaller difference between true data distribution and data distribution after deletion or imputation. M_del_: Deletion; M_fix1_: Imputation of the minimum and maximum from the measurement range for samples BC and AC, respectively; M_fix2_: Imputation of the mean between zero and lower cut-off for samples BC and the mean between upper cut-off and upper range limit for samples AC; M_fix3_: Imputation of the lower cutoff value for samples BC and the upper cut-off for samples AC; M_uni_: Imputation for samples BC and AC from a uniform distribution in the respective intervals M_log_*:* Imputation for samples BC and AC from a fitted lognormal distribution in the respective intervals.

#### 4.3.2 Trends for the standard deviation

**Fig. 5:**
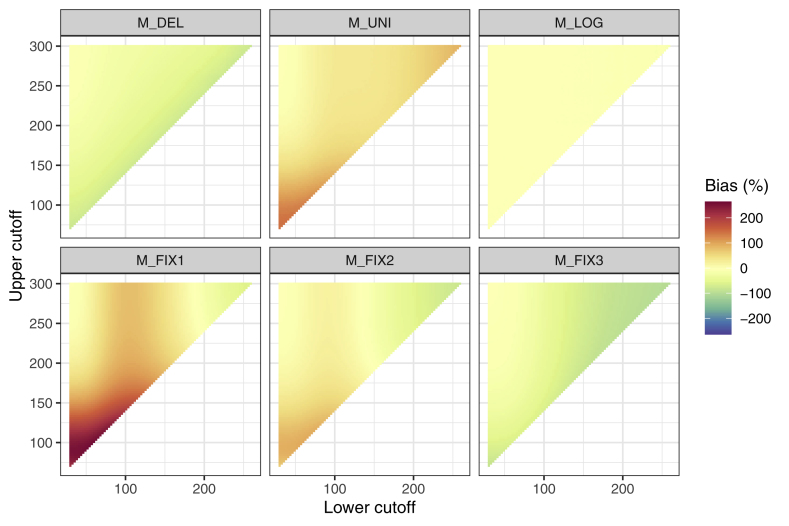
**Deviation of the of the standard deviation estimate of the reconstructed distribution compared to the standard deviation of the true distribution.** As in Fig. 4, increasing color intensity corresponds to an increasing difference of the standard deviation of the reconstructed distribution compared to the true data distribution. (For interpretation of the references to color in this figure legend, the reader is referred to the Web version of this article.)

Fig. 5 shows the results of a simulation for the difference of the standard deviation of the reconstructed distribution compared to the standard deviation of the true distribution. For M_del_ and M_fix3_, the true standard deviation is underestimated for each combination of LC and UC. The difference becomes stronger with increasing amount of censored values. M_fix1_, M_fix2_ and M_uni_ tend to overestimate the true standard deviation. This effect is particularly prominent for M_fix1_ and a large amount of censored values. Overall, the adverse effects of all methods except M_log_ are largest for a high amount of censored values. M_log_ again provides the smallest MSE (Table 3).

#### 4.3.3 Trends in the median

**Fig. 6:**
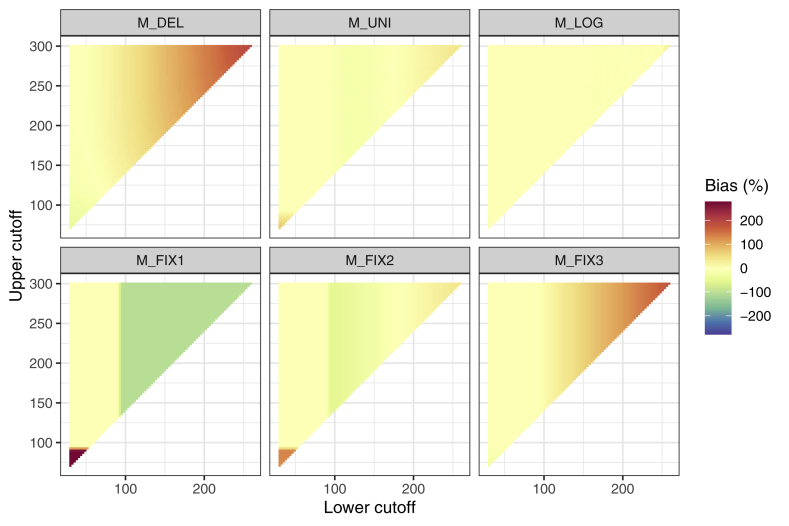
**Deviation of the median estimate of the reconstructed distribution compared to the median of the true distribution.** Note that for fixed-value imputations (M_fix1_-M_fix3_), extreme situations with more than 50% of values that one censoring cutoff lead to a median change to the imputed value of these samples. This phenomenon does not occur for distribution-based imputations (M_uni_ and M_log_).

The trend for the methods in the median estimate is illustrated in Fig. 6. The difference in the median estimate between reconstructed and true data distribution is for all methods smaller than for the mean estimate. The trend for M_del_ equals the trend for the mean estimate (4.3.1). Overall, again, the smallest differences over the applied ranges of LC and UC are observed using M_log_ (see Table 3).

#### 4.3.4 Trends for the Kolmogorov smirnov distance

**Fig. 7:**
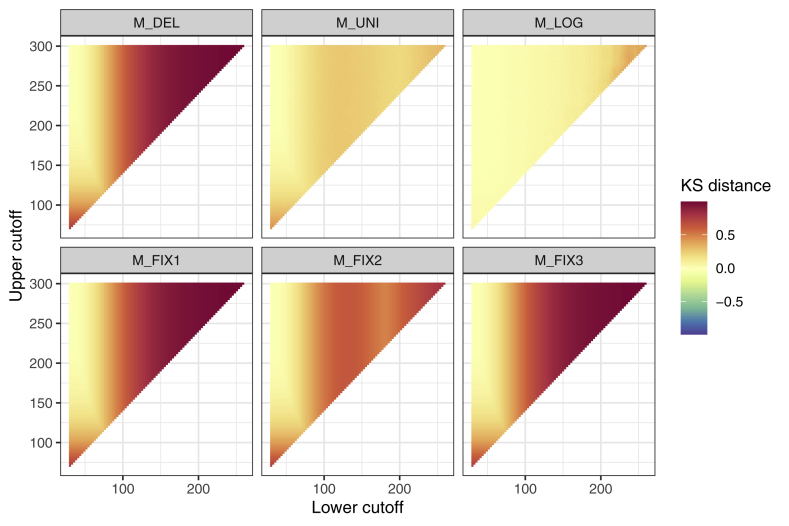
**Kolmogorov-Smirnov distance (d**_**KS**_**) of the reconstructed distribution compared to the of the true distribution.** Values can range from 0 to 1. Smaller numbers indicate a smaller distance between true data distribution and data distribution after deletion or imputation.

The effects for each method on d_KS_ are visualized in Fig. 7. For M_del_, M_fix1_, Mf_ix2_, M_fix3_ and M_uni_, the trend is towards an increasing d_KS_ both for increasing LC and UC. In other words, the deviation between the true data distribution and the data distribution after deletion or imputation progressively increases for these methods with respect to increasing LC and UC. However, this effect is notably smaller for M_uni_ than for the other four methods. No such trend is apparent for M_log_ (Table 3).

## 5 Censored regression models

In our simulations that compared the performance of deletion and methods using imputation, imputation from a fitted censored lognormal distribution has provided the most accurate parameter estimates, suggesting a valid basis for consecutive statistical analyses. We would also like to mention censored regression modeling as an advanced and powerful option to deal with ND and OV. Censored regression models, e.g. Tobit models [42], are complex statistical models which directly account for censored data, i.e. without interim steps like imputation for censored samples.

Proceeding from the measurement precision model we introduced in section 2.1, a censored regression model of biochemical data could be specified as follows: The observed data y_i_ indicates the true data y_i_∗ with adequate precision if y_i_∗ lies between an LC and UC (2). If the true data falls below LC, the sample is BC (i.e., ND) and, thus, left censored (1). Conversely, if the observed data excites UC, the sample is AC (i.e., OV) and, thus, right censored (3).

$$y_{i}=\left\{ \begin{array}{ccc} \text{BC}, & if\;y_{i}^{*}<\text{LC} & \left(1\right)\\ y_{i}^{*}, & if\;\text{BC}\leq y_{i}^{*}\leq \text{LC} & \left(2\right)\\ \text{AC} & if\;\text{UC}<y_{i}^{*} & \left(3\right) \end{array}\right.$$

y_i_ ​= ​observed value
y_i_∗ ​= ​true value
BC ​= ​below cutoff
AC ​= ​above cutoff

Censored regression models require knowledge about the mechanism that generated the observed data, that is, the position of the lower and upper censoring thresholds and accordingly the amount of BC and AC censored samples. Furthermore, model extensions allow for a modeling of covariate dependent cutoffs or multivariate dependencies, making them the method of choice for ND and OV in multivariate settings. For further information on these models and their application possibilities, we recommend the reading of Tobin [42], Amemiya [43] and Omori and Miyawaki [44].

## 6 Conclusion and recommendations

With this paper, we aimed to derive recommendations on how to deal with ND and OV in biomarker research. As missing values due to censoring are MNAR, strategies for values missing (completely) at random based on the observed values (like mean and median imputation) necessarily fail. We considered simple and common handling methods for ND and OV and introduced advanced statistical models like Tobit models that allow for a direct modeling of censored observations. In a simulation study in a univariate setting, we investigated the performance for six selected methods (deletion and five different imputation methods) in specific scenarios (with varying percentages and patterns of censored values) as well as over a broad range of censoring cutoffs. Four criteria were hereby applied: differences in mean, median and standard deviation estimates and KS distance.

Common and simple methods like a deletion of affected cases (M_del_) and fixed-value imputations (M_fix1_, M_fix2_ and M_fix3_) are a risk for biased parameter estimates, even in the presence of only low percentages of censored values. While fixed-value imputation had overall slightly preferable properties compared to deletion, both bear a high risk of systematic bias.

These results are in line with prior publications demonstrating the invalidity of deletion and different fixed-value imputations for both simulated and real data in the context of censoring [16,45]. M_del_ also leads to a loss of sample size and statistical power and as the censored cases are MNAR, its use would be statistically invalid. M_fix2_ and M_fix3_ require considerations on the (plausible) measurement range, which might be unknown and would add another free parameter to the model.

Distribution-based imputations (M_uni_ and M_log_) show a balanced profile regarding difference in mean, SD and median as well as d_KS_. Imputation based on a censored distribution fitting algorithm (M_log_) showed overall the least difference between reconstructed and true parameter estimates. In addition, d_KS_ is overall small for this method, indicating that sampling the values from the fitted intervals provides a close match of the true data distribution and the reconstructed data distribution.

Based on our simulations of the six compared methods and assumptions on the distribution characteristics of biomarker data, we therefore recommend M_log_ that uses censored regression fitting to the observed data and censored samples and impute values from the respective censored intervals. It can be implemented with the attached package for an application in R, accessible at https://osf.io/spgtv/. The present algorithm uses lognormal distribution fitting to account for the non-normal and right-skewed distribution of most biological data [30]. However, it must be mentioned that our simulations did not include a structural misspecification of the algorithm. Future research efforts will be needed to evaluate the performance of the algorithm in its present form in various contexts, for instance in the case of violated assumption for the data-generating mechanism. Hence, if there is reason to assume a different underlying distribution of the true data (e.g. in regard to the skewness of the distribution), the censored distribution fitting could be adjusted to this respective distribution. Furthermore, this article only focused on univariate settings. In cases where multiple biomarkers or repeated measures are studied, other methods might prove superior (for example, multivariate imputation using the mice package in R, see [46]).

In our view, censored regressions – as Tobit models – are likely the best option to handle ND and OV as they can model the censored distributions directly, so that no interim steps such as imputations are required. However, they are statistically complex models and their implementation might be sophisticated. We would recommend their use especially in multivariate settings or when confronted with covariate dependent cutoffs.

We would like to end our conclusion with some general recommendations and outlooks with respect to the handling and the reporting of biomarker data:

1. Knowledge on the operational range and the quantification limits of the used assay has shown to be crucial for successful identification and handling of ND and OV. Researchers should therefore seek all possible information on the measurement process from their lab if not automatically reported back (see also [26]). This should also include operational definitions of parameters, e.g. the LOD and CV ranges.
2. In each publication, information on the operational range, percentages of values above and below the quantification limits, a potential cutoff for outliers and the applied handling method should be reported. For the purposes of transparency and completeness in reporting, it should also be stated if no ND and OV were observed.
3. If ND and OV are chosen to be deleted, at least their respective percentages and the reasons that lead to this decision should be reported.
4. If ND and OV are chosen to be imputed and the required assumptions hold, we recommend using the novel algorithm M_log_. For other assumed data-generating mechanisms, the algorithm could be adjusted to the respective distribution. For distribution-based imputations, it is best practice to use multiple instead of single imputation.
5. Especially in multivariate settings or in contexts of covariate dependent quantification cutoffs, we suggest using censored regression models, for instance Tobit models.
6. Increasing the efforts in the reanalysis of samples, especially samples ND and OV, may improve the possibilities to discriminate between missing data mechanisms, i.e., MNAR and missing values due to unrelated (random) errors.

## 7 Authorship statement

All persons who meet authorship criteria are listed as authors, and all authors certify that they have participated sufficiently in the work to take public responsibility for the content, including participation in the concept, design, analysis, writing, or revision of the manuscript.

## Conflicts of interest

None.

## Declaration of interests

The authors declare that they have no known competing financial interests or personal relationships that could have appeared to influence the work reported in this paper.

## CRediT authorship contribution statement

**Judith Herbers:** Conceptualization, Methodology, Literature Review, Formal analysis, Visualization, Writing – original draft. **Robert Miller:** Conceptualization, Methodology, Technical Revision, Supervision, Writing – review & editing. **Andreas Walther:** Conceptualization, Writing – review & editing. **Lena Schindler:** Conceptualization, Writing – review & editing. **Kornelius Schmidt:** Conceptualization, Writing – review & editing. **Wei Gao:** Conceptualization, Supervision. **Florian Rupprecht:** Conceptualization, Methodology, Programming, Formal analysis, Visualization, Writing – review & editing, Judith Herbers in the name of all authors.

## Figures and Tables

**Multimedia component 1:**
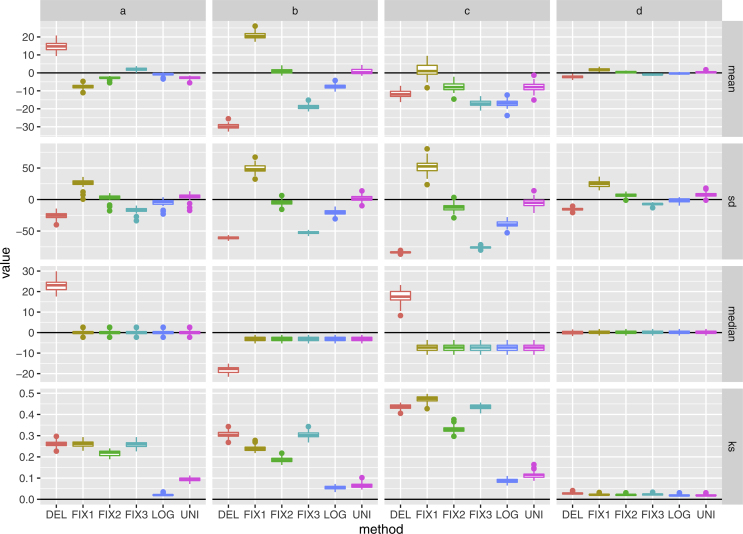


## Appendix A Supplementary data

Supplement.docx Supplement.docx

## Footnotes

1 For the purpose of this article as an easy-to-implement how-to guide, we decided to focus our presentations on univariate approaches. For the same reason, we also decided to only briefly elaborate on multiple imputation in section 3.2.2. We recommend a reading of [31,38] for more details.

2 Note that biochemical assays also have a (lower) limit of detection (LOD), which is the lowest concentration that can reliably detected, i.e., distinguished from background noise.

3 An alternative and potentially more robust approach to determine cutoffs for OV, especially in the context of skewed data distributions, may be the median absolute distance (MAD; for a more detailed consideration, also on univariate and multivariate outliers in general, see [27]).
