# Supplementary material for: How to deal with non-detectable and outlying values in biomarker research: Best practices and recommendations for univariate imputation approaches
Source: Compr Psychoneuroendocrinol. 2021 Mar 29;7:100052. doi: 10.1016/j.cpnec.2021.100052 (PMC9216349; doi:10.1016/j.cpnec.2021.100052)
Supplement: Supplement.docx [file mmc1.docx]

**How to deal with non-detectable and outlying values in biomarker research: Best practices and recommendations for univariate imputation approaches**

Herbers, Judith^1^, Miller, Robert^1,2^, Walther, Andreas^1,3^, Schindler, Lena^1^, Schmidt, Kornelius^1^, Gao, Wei^1^, Rupprecht, Florian^1^

^1^Faculty of Psychology, Technische Universität Dresden, Dresden, Germany

*²Health Technology Assessment & Outcomes Research, Health & Value, Pfizer Germany*

*³Clinical Psychology and Psychotherapy, University of Zurich, Switzerland*

**Supplementary Material**

**S1 Information on exploratory screening of methods handling non-detectable and outlying values in biomarker research**

Two coders (JH and FR) screened all open access articles published in *Psychoneuroendocrinology* in 2019 that were available on the journal website with respect to the reported practices in dealing with non-detectable (ND) and outlying values (OV). Of the 48 screened articles, 38 (79.2%) were original studies that used biomarkers. The data file and R-script for this analysis are provided on the Open Science Framework (https://osf.io/spgtv).

**ND**

Only 17 (44.7%) of the 38 biomarker studies mentioned that ND occurred. Of these, 5 (29.4%) reported deleting or excluding ND from analyses, 1 (5.9%) reported single imputation methods and 5 (29.4%) reported multivariate approaches. 6 (35.3%) did report that NDs were present but did not report how they were handled.

**OV**

13 (34.2%) of the 38 biomarker studies mentioned that OV occurred. Of these, 8 (61.5%) reported deleting or excluding OV from analyses, 1 (7.7%) reported fixed value imputation methods and 1 (7.7%) reported multivariate approaches. 3 (23.1%) did report that OV were present but did not report how they were handled. The most common reported criterion OV with 4 (30.8%) studies, was a fixed limit at a varying number of standard deviations above mean. 6 (46.2%) studies reported various other criteria, and 3 (23.1%) did not report how OV were identified.

**S2 Technical details of simulations**

To model a true data distribution, we randomly draw values from a lognormal distribution $\mathcal{LN}(\mu,\sigma^{2})$, which can be defined as

$$\mathcal{LN}(\mu,\sigma^{2})(x)=\frac{1}{\sqrt{2\pi}\sigma x}\text{exp}\left( -\frac{(\text{ln}(x)-\mu)^{2}}{2\sigma^{2}} \right) ,$$

where $x\in\mathbb{R}_{>0}$. The location parameter $\mu\in\mathbb{R}_{>0}$ and the shape parameter $\sigma\in\mathbb{R}_{>0}$ correspond to mean and standard deviation of the underlying normal distribution.

The more convenient mean $\mu'\in\mathbb{R}_{>0}$ and standard deviation $\sigma'\in\mathbb{R}_{>0}$ of the lognormal distribution can be used to calculate the shape and location parameters via

$$\mu=\text{ln}\left( \frac{{\mu'}^{2}}{\sqrt{{\sigma'}^{2}+{\mu'}^{2}}} \right)$$

and

$$\sigma=\sqrt{\text{ln}\left( 1+\frac{{\sigma'}^{2}}{{\mu'}^{2}} \right)} .$$

To model differences in the coefficient of variation

$$\mathrm{CV}(x)=a\cdot(x+b)^{2}+c ,$$

for a true value $x\in\mathbb{R}_{>0}$ and subsequently the measurement error, we use the quadratic formula where $a\in\mathbb{R}_{>0}$ is a scale parameter and $b,c\in\mathbb{R}$ are translation parameters. This function can be solved to obtain measurement range limits

$$\mathrm{CV}_{\pm}(y)=b\pm\sqrt{\frac{y-c}{a}} ,$$

for a desired maximum CV $y$.

A number of $n$ measuements

$$\hat{x}_{i}=x_{i}+\varepsilon_{i} ,$$

for $i=1,2,\ldots,n$ are simulated, where true values $x_{i}$ are randomly drawn from a lognormal distribution $\mathcal{LN}(\mu,\sigma^{2})$ and corresponding measurement errors $\varepsilon_{i}$ are randomly drawn from a normal distribution with zero mean and standard deviation $x\cdot\mathrm{CV}(x)$.

**S3 Used parameters for simulations**

**Table S1.** **Parameters for the models used in the scenarios and the range simulation.**

| Model | $n$ | $\mu'$ | $\sigma'$ | $a$ | $b$ | $c$ |
| --- | --- | --- | --- | --- | --- | --- |
| Scenario A | 1000 | 55 | 40 | 1e-05 | 100 | 0.05 |
| Scenario B | 1000 | 150 | 90 | 1e-05 | 100 | 0.05 |
| Scenario C | 1000 | 110 | 100 | 6e-05 | 100 | 0.05 |
| Scenario D | 1000 | 80 | 35 | 1e-05 | 100 | 0.05 |
| Range | 1000 | 100 | 40 | 1e-05 | 100 | 0.05 |

**S4 Visualization of the results of the scenario simulations**


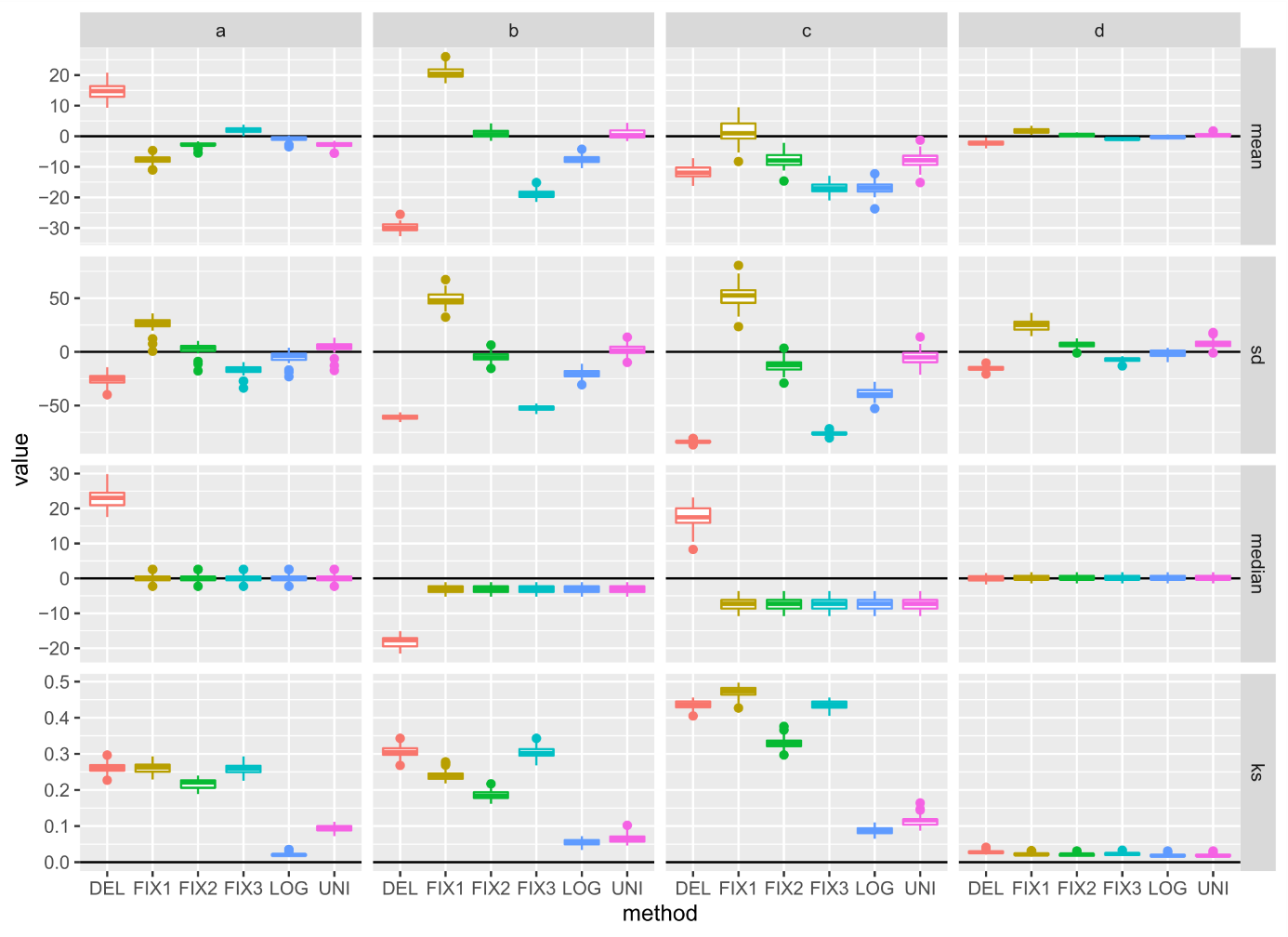


***Figure S1. Boxplots for each applied method in the established criteria in the scenarios.*** *Displayed is the deviation between reconstructed and true estimate in percent (for mean, standard deviation and median), as well as the Kolmogorov-Smirnov distance between the reconstructed and the true data distribution.*

**S5 Additional results for the simulation of varying cutoffs**

**Table S2. Additional results for the mean estimate.** Additional to the average deviations of the mean estimate which are also presented in the main text (column 1), this table includes the averaged standard deviation, minimum and maximum of the estimated deviation of the mean, as well as the average mean squared error (MSE) and the mean squared error proportional to the method deletion (M_del_; MSE_p_).

| Method | Mean | SD | Min | Max | MSE | MSEp |
| --- | --- | --- | --- | --- | --- | --- |
| M_del_ | 41.089 | 46.420 | -42.166 | 175.968 | 3842.530 | 100.000 |
| M_fix1_ | -34.892 | 47.103 | -98.934 | 179.341 | 3435.519 | 89.408 |
| M_fix2_ | -2.984 | 12.677 | -16.227 | 73.602 | 169.576 | 4.413 |
| M_fix3_ | 28.924 | 38.164 | -32.137 | 160.140 | 2292.651 | 59.665 |
| M_uni_ | -2.987 | 12.683 | -16.565 | 73.840 | 169.725 | 4.417 |
| M_log_ | 0.214 | 0.903 | -4.270 | 26.338 | 0.861 | 0.022 |

**Table S3. Additional results for the standard deviation estimate.** Additional to the average deviations of the mean estimate which are also presented in the main text (column 1), this table includes the averaged standard deviation, minimum and maximum of the estimated deviation of the standard deviation, as well as the average mean squared error (MSE) and the mean squared error proportional to the method deletion (M_del_; MSE_p_).

| Method | Mean | SD | Min | Max | MSE | MSEp |
| --- | --- | --- | --- | --- | --- | --- |
| M_del_ | -37.830 | 19.272 | -76.988 | -1.962 | 1802.441 | 100.000 |
| M_fix1_ | 70.073 | 65.014 | -56.971 | 264.069 | 9135.871 | 506.861 |
| M_fix2_ | 10.876 | 35.984 | -76.705 | 98.923 | 1412.774 | 78.381 |
| M_fix3_ | -43.665 | 28.427 | -96.144 | -0.531 | 2714.519 | 150.602 |
| M_uni_ | 42.076 | 27.146 | -0.072 | 144.723 | 2507.134 | 139.097 |
| M_log_ | -0.840 | 1.198 | -11.488 | 1.535 | 2.141 | 0.119 |

**Table S4. Additional results for the median estimate.** Additional to the average deviations of the mean estimate which are also presented in the main text (column 1), this table includes the averaged standard deviation, minimum and maximum of the estimated deviation of the median, as well as the average mean squared error (MSE) and the mean squared error proportional to the method deletion (M_del_; MSE_p_).

| Method | Mean | SD | Min | Max | MSE | MSEp |
| --- | --- | --- | --- | --- | --- | --- |
| M_del_ | 46.342 | 50.084 | -36.130 | 196.876 | 4655.227 | 100.000 |
| M_fix1_ | -49.744 | 59.437 | -100.000 | 278.353 | 6006.207 | 129.021 |
| M_fix2_ | -8.836 | 23.203 | -47.405 | 136.348 | 616.306 | 13.239 |
| M_fix3_ | 32.073 | 43.772 | -23.347 | 180.079 | 2944.111 | 63.243 |
| M_uni_ | -1.500 | 9.577 | -14.327 | 78.638 | 93.938 | 2.018 |
| M_log_ | 0.311 | 1.065 | -4.899 | 30.254 | 1.231 | 0.026 |

**Table S5. Additional results for the Kolmogorov Smirnov distance (****d_KS_).** Additional to the averaged d_KS_ which are also presented in the main text (column 1), this table includes the averaged standard deviation, minimum and maximum of the estimated deviation of d_KS_, as well as the average mean squared error (MSE) and the mean squared error proportional to the method deletion (M_del_; MSE_p_).

| Method | Mean | SD | Min | Max | MSE | MSEp |
| --- | --- | --- | --- | --- | --- | --- |
| M_del_ | 0.536 | 0.341 | 0.002 | 0.996 | 0.404 | 100.000 |
| M_fix1_ | 0.536 | 0.341 | 0.002 | 0.996 | 0.404 | 99.998 |
| M_fix2_ | 0.406 | 0.221 | 0.002 | 0.808 | 0.213 | 52.837 |
| M_fix3_ | 0.536 | 0.341 | 0.002 | 0.996 | 0.404 | 100.001 |
| M_uni_ | 0.183 | 0.087 | 0.002 | 0.444 | 0.041 | 10.165 |
| M_log_ | 0.057 | 0.067 | 0.001 | 0.427 | 0.008 | 1.919 |
